# Synthesis of Graphene Quantum Dots Enhanced Nano Ca(OH)_2_ from Ammoniated CaCl_2_

**DOI:** 10.3390/ma16041568

**Published:** 2023-02-13

**Authors:** Feng Wang, Yaoqi Gu, Jianrui Zha, Shuya Wei

**Affiliations:** Institute of Cultural Heritage and History of Science & Technology, University of Science and Technology Beijing, Beijing 100083, China

**Keywords:** Ca(OH)_2_, Graphene Quantum Dots, DFT, complex, nanoparticles

## Abstract

Ca(OH)_2_ nanoparticles are effective materials for cultural heritage restoration, hazardous substance absorption and photocatalyst. However, many methods are complex, and the particle sizes are usually above 80–100 nm, involving mediocre efficacy for application in the stone restoration field. In this work, Nano Ca(OH)_2_ with diameters less than 70 nm and composited with Graphene Quantum Dots (GQDs) were successfully synthesized in aqueous media. The morphology and structure of the nanoparticles were investigated with TEM, HRTEM, XRD, Raman and FTIR. The particle size distribution and relative kinetic stability of the Ca(OH)_2_ in ethanol were performed using a laser particle size analyzer and spectrophotometer. Firstprinciple calculations based on the spin-polarized density functional theory (DFT) were carried out to study the reaction process and combination model. The nanoparticles, as prepared, are composed of primary hexagonal crystals and high ammoniated precursors, which have a positive effect on reducing the grain size, and interacted with the GQDs hybrid process. According to the First-principle calculations results, the energy variation of the whole reaction process and the bonding mode between Ca(OH)_2_ and GQDs can be understood better.

## 1. Introduction

Nano calcium hydroxide (Nano Ca(OH)_2_), as a kind of modified lime material, is now an important chemical for hazardous substance absorption [1,2] and photocatalyst [3,4]. Thanks to its higher reactivity with CO_2_ and better penetrability, Nano Ca(OH)_2_ is an excellent consolidating material for the conservation of carbonate stones [5,6] and mural paintings [7,8] in the cultural heritage restoration field. There are two typical synthesis methods for Nano Ca(OH)_2_. One is homogeneous synthesis, using CaCl_2_ or Ca(NO_3_) [9] as calcium sources and reacting with lye in water or diol [10], and the other is the heterogeneous synthetic method, using CaO [11], CaH_2_ [12] or calcium metal [13] as precursors. However, the heterogeneous method and Ca(NO_3_) _2_ reacts violently and produces flammable and explosive products. Consequently, CaCl_2_ is the most shared reactant for Nano Ca(OH)_2_ synthesis. Furthermore, in order to improve the dispersity, short chain alcohols such as ethanol and 2-propanol are applied instead of water, resulting in more effective constituents being released in the weathered artworks [11,14,15].

Graphene Quantum Dots (GQDs), with a tiny size, primarily less than 20 nm, are described as zero-dimensional carbon materials similar to the crystalline structure of single or a few layers of graphene [16,17]. As a consequence of their unique structure, they exhibit exceptional chemical and physical properties, leading to their broad application in bioimaging, biosensing and biomedicine [18,19,20]. Quite a few functional groups, such as hydroxyl and carboxyl, comprising oxygen in the GQDs’ surface, provide good hydrophilicity and a beneficial channel for further functionalization [21]. Jinmeng Zhu et al. obtained graphene-enhanced Nano Ca(OH)_2_ around 80 nm in size and achieved good results on wall painting protection [22]. GQDs are considered to be inhibitors of growth, which may be attributed to an effect of the surfactant and increased supersaturation degree of the reaction solution [23]. However, for marble relics, a multitude of pores below 80 nm in the host rock can be detected using the mercury intrusion method [24]; meanwhile, salt crystallization-related rock degradation is most significantly influenced by tiny pores [25]. Therefore, smaller Ca(OH)_2_ nanoparticles are expected to be used for stone restoration and the effect of GQDs needs more investigation.

NH_3_ can be absorbed by CaCl_2_ and forms a complex of metal, as shown in the chemical Equations (1)–(3), where ∆H_1_, ∆H_2_, and ∆H_3_ are the enthalpies of the transformation for the reactions (J/mol), and T_e1_, T_e2_, and T_e3_ are the equivalent temperatures for the reactions. In exothermic reactions, the potential energy decreases to a low value when the chemical adsorption is stable [26]. It is not easy to release the last ammonia molecule of a complex [27], leading to a higher crystallization energy barrier for Ca(OH)_2_ and, hopefully, obtaining smaller crystals.

(1) $${\mathrm{CaCl}}_{2}+2{\mathrm{NH}}_{3}\;↔{\mathrm{CaCl}}_{2}\cdot 2{\mathrm{NH}}_{3}+\Delta H_{1}{\;\;\;\;\;\mathrm{at}\;\mathrm{Temperature}\;T}_{e1}$$

(2) $${\mathrm{CaCl}}_{2}\cdot 2{\mathrm{NH}}_{3}+2{\mathrm{NH}}_{3}\;↔{\;\mathrm{CaCl}}_{2}\cdot 4{\mathrm{NH}}_{3}+\Delta H_{2}{\;\;\mathrm{at}\;\mathrm{Temperature}\;T}_{e2}$$

(3) $${\mathrm{CaCl}}_{2}\cdot 4{\mathrm{NH}}_{3}+4{\mathrm{NH}}_{3}↔{\mathrm{CaCl}}_{2}\cdot 8{\mathrm{NH}}_{3}+\Delta H_{3}{\;\;\;\;\mathrm{at}\;\mathrm{Temperature}\;T}_{e3}$$

In this study, ammoniated CaCl_2_ was used instead of CaCl_2_ as the calcium precursor to synthesize smaller Nano Ca(OH)_2_, while modifying GQDs to enhance the nanoparticles. Different molar equivalences of ammonia were explored and we characterized their morphology, particle size and structure features. The relative kinetic stability in ethanol was investigated. The whole reaction process and the possible combination mode were performed using first-principle calculations based on the Density Functional Theory (DFT) for better understanding.

## 2. Materials and Methods

### 2.1. Materials

CaCl_2_·2H_2_O (≥99.0%), NaOH (≥96.0%), Ca(OH)_2_ (≥95.0%)and ethanol (≥99.7%) were obtained from Sinopharm Chemical Reagent Co. LTD. The ammonia solution (Ammonia 20%) and AgNO_3_ (≥99.8%) were purchased from Shanghai Aladdin Bio-Chem Technology Co.,LTD. The Graphene Quantum Dots solution (0.10 mg/mL) were supplied by Yan Li’s lab through the cyclic voltammograms method [28].

### 2.2. Synthesis of GQDs Enhanced Nano Ca(OH)_2_

In order to obtain the CaCl_2_ solution, 14.7 g CaCl_2_·2H_2_O was dissolved in 500 mL deionized water; then, 0 mol, 0.2 mol, 0.4 mol or 0.8 mol ammonia was added, respectively, after being heated to 80 °C in a glass agitated reactor, named the C-A solution. Next, 8.0 g NaOH and 10 mL GQDs solution were added into 200 mL deionized water and heated to 80 °C in a water bath kettle, named the N-G solution. After stirring for 60 min, the N-G solution was dropped into the C-A solution at a rate of 100 mL/min. The rate of agitation should be above 350 rpm. The reactor was removed from the heat and sealed immediately. The water in the bath kettle of the reactor was changed for quick cooling to room temperature. Saturated Ca(OH)_2_ solution was used for washing by centrifugal, until no chloride ion could be detected by 1 mol/L AgNO_3_ solution. After drying for 12 h at 50 °C in a vacuum drying chamber, 0.2 g powder was dispersed in 200 mL ethanol for further characterization. The products prepared with CaCl_2_, CaCl_2_·2NH_3_, CaCl_2_·4NH_3_ and CaCl_2_·8NH_3_ as precursors were named A1, A2, A3 and A4, respectively.

### 2.3. Characterization of Nano Ca(OH)_2_

Transmission electron microscopy (TEM) images were recorded on a JEOL JEM1200EX (JEOL Ltd., Tokyo, Japan) electron microscope at 100 kV. High resolution transmission electron microscopy (HRTEM) photographs and selected-area electron diffraction (SAED) were performed on a FEI TECNAI G2 F30 (FEI company, Hillsboro, USA) microscope operating at 300 kV. The Gatan Digital Micrograph (DM) software was used to analyze the crystal planes and SAED data. The particle size distribution was taken by the Malvern Zetasizer Nano ZS90 (Malvern Ltd., Malvern, UK) laser particle size analyzer (PSA). X-ray diffraction (XRD) was operated with Rigaku Smartlab (Rigaku Corporation, Tokyo, Japan) at 40 kV, 30 mA and CuKα radiation, range of 10–90° 2θ and 0.02° 2θ step size. After collecting the data, the Rietveld method was carried out to quantify the phase compositions of the stones. The Raman spectra were measured with a HORIBA Scientific XploRA PLUS (HORIBA FRANCE SAS, Palaiseau, France) spectrometer at room temperature, using 532 nm laser excitation, Olympus 50X LWD (Olympus Corporation, Tokyo, Japan) visible objective. The infrared spectra were obtained with ThermoFisher Nicolet IS10 (Thermo Fisher Scientific, Waltham, USA) Fourier Transform Infrared Spectroscopy (FTIR) at 0.4 cm^−1^ resolution. The relative kinetic stability of Nano Ca(OH)_2_ in ethanol was evaluated by the ξ parameter (Equation (4)) through measuring the absorbance at 600 nm with Shimadzu UV-3600 (Shimadzu Corporation, Kyoto, Japan) spectrophotometer for 24 h [29,30].

ξ = {1 − [(A_t = 0_ − A_t_)/A_t = 0_]} × 100 (4)

where A_t = 0_ is the absorbance of the dispersion immediately after 15 min ultrasound, A_t_ is the absorbance at t time.

### 2.4. First-Principle Calculations Based on DFT

All of the calculations were carried out by means of spin-polarized density functional theory (DFT) methods using the Vienna Ab-initio Simulation Package (VASP) [31,32]. The exchange and correlation energies were described by the generalized gradient approximation (GGA) with the Perdew-Burke-Ernzerhof (PBE) function [33]. The projector augmented-wave (PAW) method was used to describe the electron-ion interactions [34]. To accurately describe the dispersion interaction, we used the DFT-D3 method with Becke-Jonson damping for dispersion correction [35]. A Gaussian smearing of 0.05 eV was applied to speed up the electronic convergence. The Brillouin zone was sampled with a k-point spacing of 0.06 Å^−1^ mesh [36]. The convergence tolerance of the electronic structure and geometry optimization was 1 × 10^−4^ eV, 0.05 eV/Å, respectively. The plane wave cut-off was set to 400 eV. A vacuum height of 15 Å along the vertical direction was selected to avoid the unwanted interaction between the slab and its period images. The geometrical configurations and charge density difference plots were illustrated with the VESTA software [37].

## 3. Results and Discussion

### 3.1. Morphology and Particle Size Distribution

**Figure 1:**
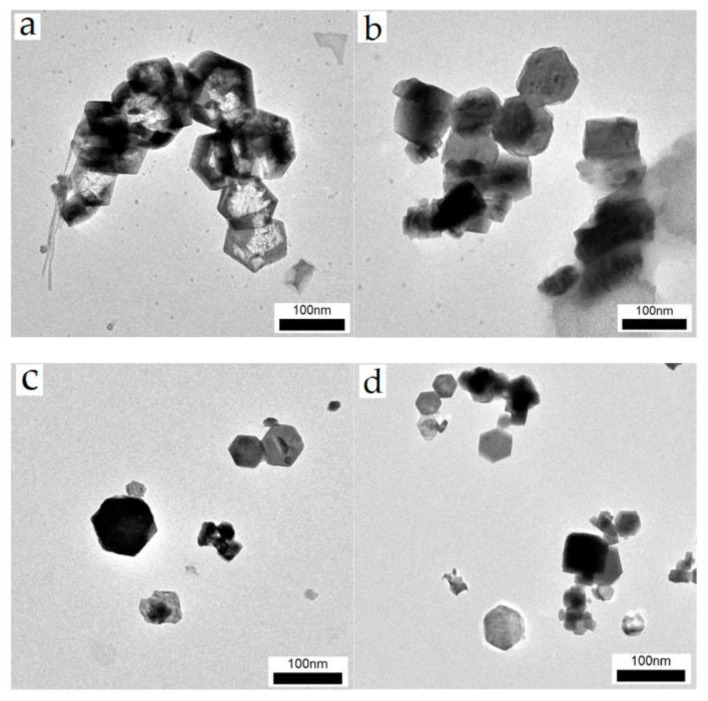
TEM images of as prepared samples ((**a**). A1; (**b**). A2; (**c**). A3; (**d**). A4).

**Figure 2:**
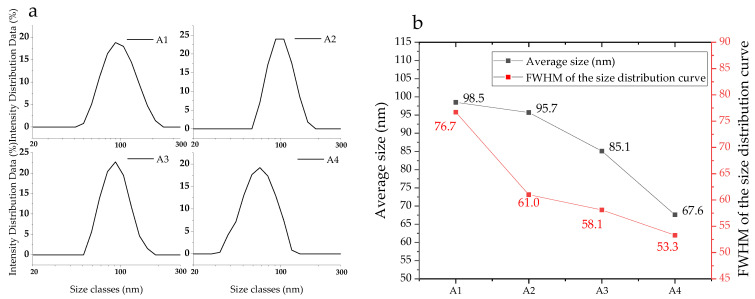
Particle size distribution of the nanoparticles (**a**) and the average size and FWHM of the size distribution curve (**b**).

Figure 1 illustrates the morphology of the synthetic Nano Ca(OH)_2_, whose dispersions in ethanol are oyster white (Figure S1, Supplementary Material). Calcium hydroxide crystals of hexagonal plates and calcium carbonate crystals of tetrahedron [38] can be found, while the particle sizes ranged between 50 nm to 110 nm. The A1−A2 samples (Figure 1a,b) are prone to aggregate; additionally, a host of tiny particles are displayed in the field of view, owing to unreacted GQDs. In contrast, the tiny matters are not present in A3 (Figure 1c) or A4 images (Figure 1d), which is likely because the hybrid proportion of GQDs is higher than A1 and A2. From the particle size analysis results, demonstrated in Figure 2, the size distributions of the as prepared nanoparticles are narrow and become more concentrated, according to the FWHM variation (red curve in Figure 2b), with a reduced average size from A1 (98.5 nm) to A4 (67.6 nm). In the reaction system of the high ammoniated precursors, particularly the octahedral one, the particle size exhibits a significant decrease, which is probably due to the GQDs hybrid and high crystallization energy barrier.

**Figure 3:**
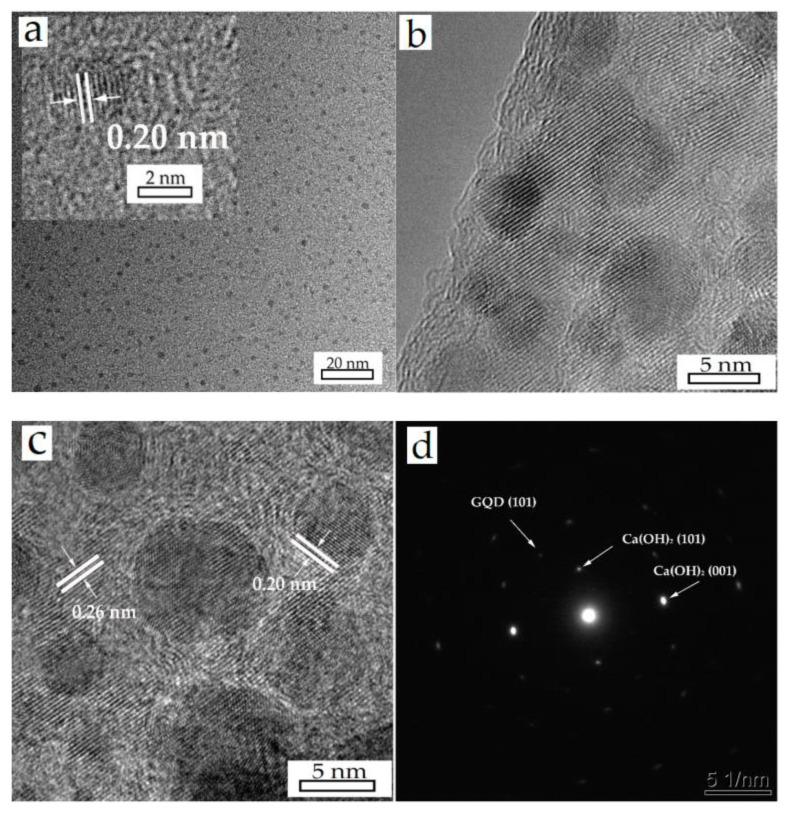
HRTEM images of GQDs (**a**) and A4 (**b**,**c**) sample, (**d**) is the SAED result of the prepared Ca(OH)_2_/GQDs composite.

Figure 3a exhibits the high-resolution photographs of the GQDs. GQDs have spherical structures and are well-dispersed, varying between 1 nm to 5 nm with about 3 nm average size. The lattice parameter was measured to be 0.20 nm, matching with the (101) plane of graphite (PDF#41−1487). The HRTEM images, presented in Figure 3b,c, show the morphology of a hybrid nanoparticle’s edge and center, from which we can estimate that the GQDs are almost monodispersed on the surface. Lattice fringes of 0.26 nm are indexed to the (101) plane of Ca(OH)_2_ (PDF#81−2040) and the same lattice fringes of 0.20 nm are assigned to the GQDs. For further confirmation, the (101) and (001) planes of Ca(OH)_2_, along with the (101) plane of the GQDs can be identified in the SAED result, displayed in Figure 3d.

### 3.2. Composition of the Materials

**Figure 4:**
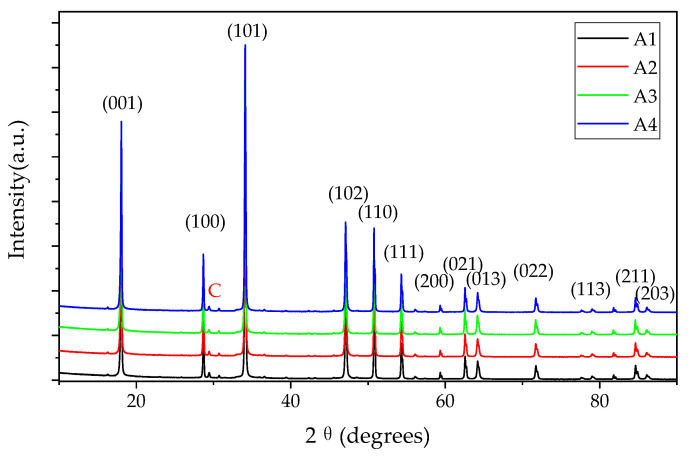
XRD patterns of the samples (calcium hydroxide: PDF#81−2040, C: Calcite, PDF#72−1652).

**Figure 5:**
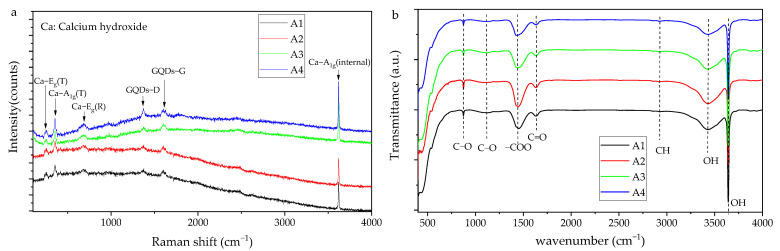
Raman spectra (**a**) and FTIR results (**b**) of the Ca(OH)_2_ powders.

The XRD patterns of the samples are shown in Figure 4. The comparison with the ICSD reference database indicates that all the powders are mainly calcium hydroxide (PDF#81−2040) and handful calcite (PDF#72−1652). The XRD pattern of the GQDs is not found, such as the (002) plane located at 26.3° [39], as a consequence of the low content and not being crystalline. Raman and FTIR were applied to obtain more structure information, as shown in Figure 5. The Raman characteristic peaks of Ca(OH)_2_ are E_g_(T) 253.7 cm^−1^, A_1g_(T) 358.1 cm^−1^, E_g_(R) 681.5 cm^−1^ and A_1g_(internal) 3620.1 cm^−1^ [40], which could be identified in the Raman results (Figure 5a). The Raman spectra of A1 to A4 also have the D-band (1368.1 cm^−1^) and G-band (1597.0 cm^−1^) and their I_D_/I_G_ are around 0.45, close to Yan Li’s results [28]. The positions of the Raman bands do not have a significant difference between the samples with different particle sizes. It is probable that the powders of the Ca(OH)_2_ possess massive aggregation after drying and the crystallinity of the particles is similar, according to the XRD results. In Figure 5b, the stretching vibration at 875 cm^−1^ is ascribed to the C−O group of calcites, resulting from the carbonation effect during the FTIR test. The peaks at 1127 cm^−1^, 1427 cm^−1^, 1633 cm^−1^ and 2926 cm^−1^ are assigned to the C−O, −COO, C=O and C−H groups on GQDs, respectively. The stretching vibrations at 3431 cm^−1^, 3642 cm^−1^ are attributed to −OH groups from Ca(OH)_2_. The N−H group is not observed, illustrating that ammonia do not combine with Ca(OH)_2_ or GQDs. No new band or evident peak shift are identified in the XRD, Raman and FTIR results, demonstrating that it is unlikely that Ca(OH)_2_ and GQDs are combined through a chemical bond.

### 3.3. Stability of Dispersions

**Figure 6:**
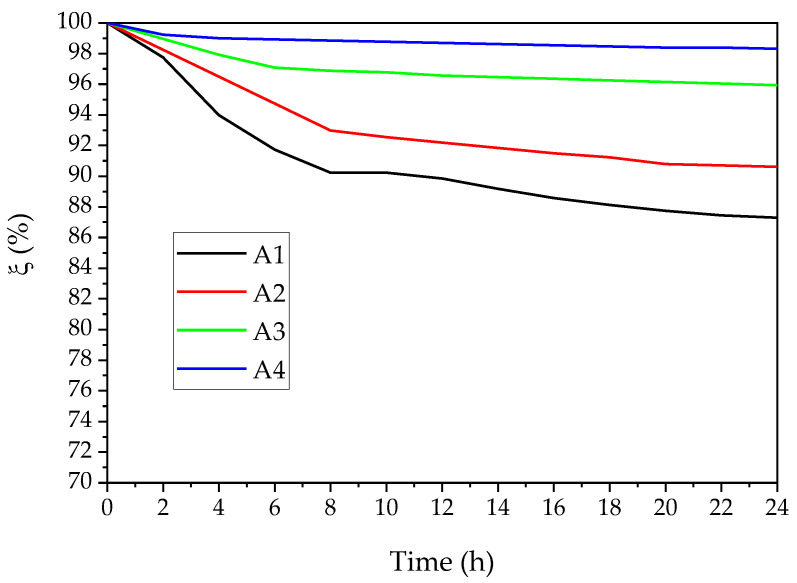
Colloidal relative kinetic stability of Nano Ca(OH)_2_ in ethanol (concentration: 1.0 g/L).

The ξ parameter at 600 nm was calculated to evaluate the kinetic stability of Nano Ca(OH)_2_ in ethanol, as is revealed in Figure 6. The A1 samples, which were synthesized without an ammino complexation, indicate a distinct decrease during the first 8 h, then a slight rise at 24 h. The A2 dispersion shows a similar trend to A1, while the stability is better. A3 and A4 present good kinetic stability, particularly A4 can keep stable just after 2 h and remain above 98 throughout the test. According to the steric stabilization [41,42], the adsorption of the ethanol molecules and GQDs on the Ca(OH)_2_ nanoparticles’ surface is helpful to hinder the stabilization of the colloidal dispersion; the smaller crystal size of A4 also serves the synergistic effect.

### 3.4. First-Principle Calculations of the Reaction and Combination

**Figure 7:**
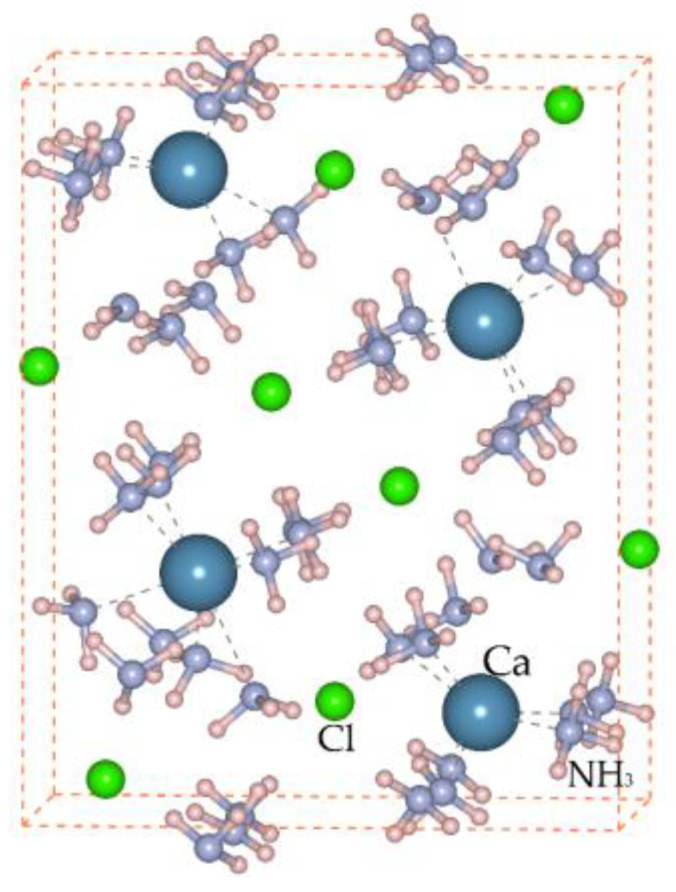
Unit cell of CaCl_2_·8NH_3_.

There are two important reactions during the whole process, as shown in Equations (5) and (6). The unit cell of CaCl_2_·8NH_3_ for the reaction energy calculation is depicted in Figure 7, and Equation (7) describes the calculation procedure. The ΔE of the transformation process from CaCl_2_ to CaCl_2_·8NH_3_ (Equation (5); see details in Table S1, Supplementary Material) is −2.0 eV, indicating an exothermic reaction. The ΔE of the whole chemical process (Equation (6); see details in Table S2, Supplementary Material) is −1.26 eV, suggesting a spontaneous chemical reaction accompanied with an exothermic nature, according to the chemical thermodynamics theory [43].

(5) $${\mathrm{CaCl}}_{2}+8{\mathrm{NH}}_{3}\cdot H_{2}O={\mathrm{CaCl}}_{2}\cdot 8{\mathrm{NH}}_{3}+8H_{2}O$$

(6) $${\mathrm{CaCl}}_{2}\cdot 8{\mathrm{NH}}_{3}+2\mathrm{NaOH}+8H_{2}O+\mathrm{GQDs}=\mathrm{Ca}{\left(\mathrm{OH}\right)}_{2}\&\mathrm{GQDs}+2\mathrm{NaCl}+8{\mathrm{NH}}_{3}\cdot H_{2}O$$

(7) $$\Delta E=\frac{E_{p}-E_{r}}{S_{\mathrm{Ca}}}$$

where ΔE is the reaction energy, E_p_ is the ground-state energy of the product, E_r_ is the ground-state energy of the reactant, S_ca_ is the stoichiometric number of the Ca atom for each unit cell.

**Figure 8:**
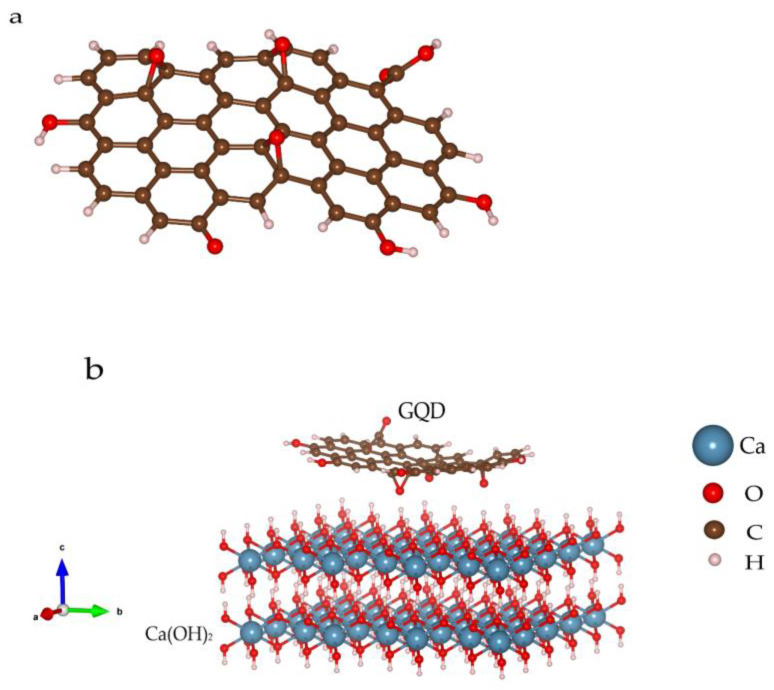
GQD molecule configuration (**a**) and combination mode between GQD and Ca(OH)_2_ (**b**).

An 11 × 17 Å^2^ GQD molecule was established, and C−H, C−O−C and C−OH bonds were modified on the edge and surface (Figure 8a), owing to the FTIR and Yan Li’s results [28]. The bulk Ca(OH)_2_ supercell of the 8169 Å^3^ volume was set up for the combination simulation, while the projection vector of the Ca(OH)_2_ lattice planes is (001), as shown in Figure 8b. According to the calculation results, the oxygen atoms on the GQDs’ surfaces are prone to form hydrogen bonds with hydroxyl from Ca(OH)_2_. The binding energy, based on Equation (8), is −11.55 meV/atom (see details in Table S3, Supplementary Material), the negative value of which indicates a more stable structure through the combination. Furthermore, no strong chemical bond will form considering the calculation results, which is identified with the structure analysis and HRTEM data.

(8) $$\Delta E_{b}=\frac{E\left(\mathrm{GQD}+\mathrm{substrate}\right)-E\left(\mathrm{GQD}\right)-E\left(\mathrm{substrate}\right)}{N\left(\mathrm{GQD}\right)}$$

where ΔE_b_ is the binding energy, N(GQD) is the number of atoms in each GQD.

## 4. Conclusions

GQDs-enhanced hexagonal Ca(OH)_2_ nanoparticles were successfully prepared using ammoniated CaCl_2_ as a precursor in aqueous media. Through TEM and PSA characterization, the average sizes decreased from 98.5 nm to 67.6 nm, benefitting from high ammoniated precursors and the GQDs hybrid. The results of the XRD, Raman and FTIR analysis show the distinct structure of the Ca(OH)_2_ and GQDs, and no new band or significant peak shift were found, indicating that they are not likely to have been coupled chemically. Synthetic nanomaterials have good dispersibility in alcohol thanks to the adsorption of ethanol molecules and GQDs on the Ca(OH)_2_ surface. The first-principle calculation results, based on the DFT, indicate the ammonia complex formation in the aqueous, and the synthesis of the Ca(OH)_2_/GQDs composite, are both spontaneous chemical reactions accompanied with an exothermic nature. The oxygen atoms on GQDs are prone to form hydrogen bonds, not chemical bonds, with Ca(OH)_2_ and the binding energy is −11.55 meV/atom, suggesting a more stable structure of the hybrid material. Combined with the HRTEM and SAED data, GQDs are certainly attached to the Ca(OH)_2_ surface through this method, which offers a new thought for the synthesis of smaller nanoparticles.

## Data Availability

The data presented in this study are available upon request from the corresponding author. The data are not publicly available due to privacy.

## Supplementary Materials

The following supporting information can be downloaded at: https://www.mdpi.com/article/10.3390/ma16041568/s1, Figure S1: Photograph of prepared samples; Table S1: Ground-state energy and the Energy contribution of each chemical in Equation (5); Table S2: Ground-state energy and the Energy contribution of each chemical in Equation (6); Table S3: Ground-state energy of each chemical for binding energy calculation in Equation (8).

## References

[1] Du Y., Meng Q., Hou R., Yan J., Dai H., Zhang T. (2012). Fabrication of nano-sized Ca(OH)_2_ with excellent adsorption ability for N_2_O_4_. Particuology.

[2] Mohd Daud F.D., Vignesh K., Sreekantan S., Mohamed A.R., Kang M., Kwak B.S. (2016). Ca(OH)_2_ nano-pods: Investigation on the effect of solvent ratio on morphology and CO_2_ adsorption capacity. RSC Adv..

[3] Zhang S. (2014). A new nano-sized calcium hydroxide photocatalytic material for the photodegradation of organic dyes. RSC Adv..

[4] Narayan R.B., Goutham R., Srikanth B., Gopinath K.P. (2018). A novel nano-sized calcium hydroxide catalyst prepared from clam shells for the photodegradation of methyl red dye. J. Environ. Chem. Eng..

[5] Daniele V., Taglieri G. (2011). Ca(OH)_2_ nanoparticle characterization: Microscopic investigation of their application on natural stones. WIT Trans. Eng. Sci..

[6] Caner E., Caner-Saltık E.N. (2018). A practical method for preparing Ca(OH)_2_ nanodispersions for the consolidation of archaeological calcareous stones. Mediterr. Archaeol. Archaeom..

[7] Baglioni P., Chelazzi D., Giorgi R., Carretti E., Toccafondi N., Jaidar Y. (2013). Commercial Ca(OH)_2_ nanoparticles for the consolidation of immovable works of art. Appl. Phys. A.

[8] Chelazzi D., Poggi G., Jaidar Y., Toccafondi N., Giorgi R., Baglioni P. (2013). Hydroxide nanoparticles for cultural heritage: Consolidation and protection of wall paintings and carbonate materials. J. Colloid Interface Sci..

[9] Roy A., Bhattacharya J. (2010). Synthesis of Ca(OH)_2_ nanoparticles by wet chemical method. Micro Nano Lett..

[10] Salvadori B., Dei L. (2001). Synthesis of Ca(OH)_2_ Nanoparticles from Diol. Langmuir.

[11] Rodriguez-Navarro C., Suzuki A., Ruiz-Agudo E. (2013). Alcohol Dispersions of Calcium Hydroxide Nanoparticles for Stone Conservation. Langmuir.

[12] Delfort B., Born M., Chivé A., Barré L. (1997). Colloidal calcium hydroxide in organic medium: Synthesis and analysis. J. Colloid Interface Sci..

[13] Poggi G., Toccafondi N., Chelazzi D., Canton P., Giorgi R., Baglioni P. (2016). Calcium hydroxide nanoparticles from solvothermal reaction for the deacidification of degraded waterlogged wood. J. Colloid Interface Sci..

[14] Baglioni M., Poggi G., Chelazzi D., Baglioni P. (2021). Advanced materials in cultural heritage conservation. Molecules.

[15] Natali I., Saladino M.L., Andriulo F., Martino D.C., Caponetti E., Carretti E., Dei L. (2014). Consolidation and protection by nanolime: Recent advances for the conservation of the graffiti, Carceri dello Steri Palermo and of the 18th century lunettes, SS. Giuda e Simone Cloister, Corniola (Empoli). J. Cult. Heritage.

[16] Bacon M., Bradley S.J., Nann T. (2014). Graphene quantum dots. Part. Part. Syst. Charact..

[17] Tabish T.A., Zhang S. (2016). Graphene quantum dots: Syntheses, properties, and biological applications. Comprehensive Nanoscience and Nanotechnology.

[18] Chung S., Revia R.A., Zhang M. (2021). Graphene quantum dots and their applications in bioimaging, biosensing, and therapy. Adv. Mater..

[19] Zhao J., Chen G., Zhu L., Li G. (2011). Graphene quantum dots-based platform for the fabrication of electrochemical biosensors. Electrochem. Commun..

[20] Markovic Z.M., Ristic B.Z., Arsikin K.M., Klisic D.G., Harhaji-Trajkovic L.M., Todorovic-Markovic B.M., Kepic D.P., Kravic-Stevovic T.K., Jovanovic S.P., Milenkovic M.M. (2012). Graphene quantum dots as autophagy-inducing photodynamic agents. Biomaterials.

[21] Zheng X.T., Ananthanarayanan A., Luo K.Q., Chen P. (2015). Glowing graphene quantum dots and carbon dots: Properties, syntheses, and biological applications. Small.

[22] Zhu J., Li X., Zhang Y., Wang J., Wei B. (2018). Graphene-Enhanced Nanomaterials for Wall Painting Protection. Adv. Funct. Mater..

[23] Baglioni P., Chelazzi D., Giorgi R., Baglioni P., Chelazzi D., Giorgi R. (2015). Consolidation of Wall Paintings and Stone. Nanotechnologies in the Conservation of Cultural Heritage: A Compendium of Materials and Techniques.

[24] Sassoni E., Franzoni E. (2014). Influence of porosity on artificial deterioration of marble and limestone by heating. Appl. Phys. A.

[25] Yu S., Oguchi C.T. (2009). Complex relationships between salt type and rock properties in a durability experiment of multiple salt–rock treatments. Earth Surf. Process. Landf..

[26] Wang L., Wang R., Wu J., Wang K. (2005). Research on the chemical adsorption precursor state of CaCl_2_−NH_3_ for adsorption refrigeration. Sci. China Ser. E Technol. Sci..

[27] Donkers P.A.J. (2015). Experimental Study on Thermochemical Heat Storage Materials.

[28] Li Y., Liu H., Liu X.-Q., Li S., Wang L., Ma N., Qiu D. (2016). Free-radical-assisted rapid synthesis of graphene quantum dots and their oxidizability studies. Langmuir.

[29] Esumi K., Takamine K., Ono M., Osada T., Ichikawa S. (1993). The interaction of poly (vinylpyrrolidone) and solid particles in ethanol. J. Colloid Interface Sci..

[30] Giorgi R., Dei L., Baglioni P. (2000). A new method for consolidating wall paintings based on dispersions of lime in alcohol. Stud. Conserv..

[31] Kresse G., Joubert D. (1999). From ultrasoft pseudopotentials to the projector augmented-wave method. Phys. Rev. B.

[32] Kresse G., Furthmüller J. (1996). Efficient iterative schemes for ab initio total-energy calculations using a plane-wave basis set. Phys. Rev. B.

[33] Perdew J.P., Burke K., Ernzerhof M. (1996). Generalized gradient approximation made simple. Phys. Rev. Lett..

[34] Blöchl P.E. (1994). Projector augmented-wave method. Phys. Rev. B.

[35] Grimme S., Ehrlich S., Goerigk L. (2011). Effect of the damping function in dispersion corrected density functional theory. J. Comput. Chem..

[36] Methfessel M., Paxton A.T. (1989). High-precision sampling for Brillouin-zone integration in metals. Phys. Rev. B.

[37] Momma K., Izumi F. (2011). VESTA 3 for three-dimensional visualization of crystal, volumetric and morphology data. J. Appl. Crystallogr..

[38] Mercedes-Martín R., Rogerson M., Prior T.J., Brasier A.T., Reijmer J.J., Billing I., Matthews A., Love T., Lepley S., Pedley M. (2021). Towards a morphology diagram for terrestrial carbonates: Evaluating the impact of carbonate supersaturation and alginic acid in calcite precipitate morphology. Geochim. Cosmochim. Acta.

[39] Wang L., Wang Y., Xu T., Liao H., Yao C., Liu Y., Li Z., Chen Z., Sun L., Wu M. (2014). Gram-scale synthesis of single-crystalline graphene quantum dots with superior optical properties. Nat. Commun..

[40] Padanyi Z.V. (1970). The Raman spectrum of Ca(OH)_2_. Solid State Commun..

[41] Liz-Marzán L.M., Correa-Duarte M.A., Pastoriza-Santos I., Mulvaney P., Ung T., Giersig M., Kotov N.A. (2001). Core-shell nanoparticles and assemblies thereof. Handbook of Surfaces and Interfaces of Materials.

[42] Ambrosi M., Dei L., Giorgi R., Neto C., Baglioni P. (2001). Stable dispersions of Ca(OH)_2_ in aliphatic alcohols: Properties and application in cultural heritage conservation. Trends Colloid Interface Sci. XV.

[43] Smith E.B. (2004). Basic Chemical Thermodynamics.

