# Supplementary material for: Synthesis of Graphene Quantum Dots Enhanced Nano Ca(OH)2 from Ammoniated CaCl2
Source: Materials (Basel). 2023 Feb 13;16(4):1568. doi: 10.3390/ma16041568 (PMC9967000; doi:10.3390/ma16041568)
Supplement: Supplementary file 1 [file materials-16-01568-s001.zip › materials-2194062-supplementary.pdf]

Article

Synthesis of Graphene Quantum Dots Enhanced Nano $Ca(OH)_2$ from ammoniated $CaCl_2$

Feng Wang *, Yaoqi Gu, Jianrui Zha and Shuya Wei *

Institute of Cultural Heritage and History of Science & Technology, University of Science and Technology Beijing, Beijing 100083, China
* Correspondence: wangfeng1911@126.com (F.W.); swei@ustb.edu.cn (S.W.)

Supplementary Material

3.1 Morphology and Particle Size Distribution

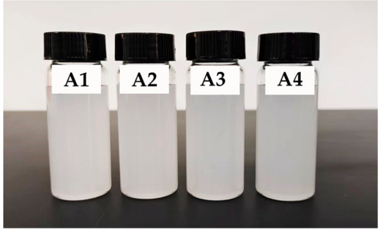

Figure S1. Photograph of prepared samples

3.4. First-Principle Calculations of the Reaction and Combination

Table S1. Ground-state energy and the Energy contribution of each chemical in Equation (5)

Chemicals	Ground-state energy (eV)	Energy contribution in reaction (eV)
$CaCl_2$	−25.62	−51.25
$NH_3{\cdot}H_2O$	−34.05	−1089.68
$CaCl_2{\cdot}(NH_3)_8$	−686.78	−686.78
H_2O	−14.32	−458.15

Table S2. Ground-state energy and the Energy contribution of each chemical in Equation (6)

Chemicals	Ground-state energy (eV)	Energy contribution in reaction (eV)
$CaCl_2{\cdot}(NH_3)_8$	−686.78	−686.78
NaOH	−27.28	−109.12
H_2O	−14.32	−458.15
$Ca(OH)_2$	−28.65	−57.30
NaCl	−27.11	−108.45
$NH_3{\cdot}H_2O$	−34.05	−1089.68

Table S3. Ground-state energy of each chemical for binding energy calculation in Equation (8)

Chemicals	Ground-state energy （eV）
GQDs	−596.31
$Ca(OH)_2$-(001)	−1367.02
$Ca(OH)_2$/GQDs	−1964.24
